# Validate your white matter tractography algorithms with a reappraised ISMRM 2015 Tractography Challenge scoring system

**DOI:** 10.1038/s41598-023-28560-w

**Published:** 2023-02-09

**Authors:** Emmanuelle Renauld, Antoine Théberge, Laurent Petit, Jean-Christophe Houde, Maxime Descoteaux

**Affiliations:** 1 Sherbrooke Connectivity Imaging Laboratory (SCIL), Computer Sciences Department, Université de Sherbrooke, Sherbrooke, Canada; 2 Université de Bordeaux, CNRS, CEA, IMN, GIN, UMR 5293, 33000 Bordeaux, France; 3 Imeka Solutions Inc, Sherbrooke, QC Canada

**Keywords:** Computer science, Imaging techniques, 3-D reconstruction, Diffusion tensor imaging, Magnetic resonance imaging

## Abstract

Since 2015, research groups have sought to produce the ne plus ultra of tractography algorithms using the ISMRM 2015 Tractography Challenge as evaluation. In particular, since 2017, machine learning has made its entrance into the tractography world. The ISMRM 2015 Tractography Challenge is the most used phantom during tractography validation, although it contains limitations. Here, we offer a new scoring system for this phantom, where segmentation of the bundles is now based on manually defined regions of interest rather than on bundle recognition. Bundles are now more reliably segmented, offering more representative metrics for future users. New code is available online. Scores of the initial 96 submissions to the challenge are updated. Overall, conclusions from the 2015 challenge are confirmed with the new scoring, but individual tractogram scores have changed, and the data is much improved at the bundle- and streamline-level. This work also led to the production of a ground truth tractogram with less broken or looping streamlines and of an example of processed data, all available on the Tractometer website. This enhanced scoring system and new data should continue helping researchers develop and evaluate the next generation of tractography techniques.

## Introduction

Tractography allows for the in-vivo non-invasive recovery of white-matter fiber trajectories in the brain. A good tractography algorithm builds a tractogram (set of streamlines) representing the ground truth (GT) of the brain anatomy. But such a GT still does not exist for in vivo data^1,2^. To alleviate this limitation and enable the evaluation of tractography algorithms’ quality, one typically relies on phantoms: simulated diffusion-weighted images (DWI) associated with GT tractograms^1^. The level of similarity between an output tractogram and the GT can be scored based on various metrics such as false positive/false negative rates, or coverage metrics such as overlap or overreach, amongst others^3^. A tractogram is generally not scored at the level of individual streamlines but rather with respect to the underlying GT bundles of the phantom. Hence, the first step in scoring the tractogram is bundle segmentation, namely assigning each streamline to a single GT bundle (or defining it as an invalid streamline). A phantom must thus be associated with a scoring system of its own, including a process for bundle segmentation and metrics that quantify the quality of these bundles. In the case of the ISMRM 2015 Tractography Challenge^4^, during which 20 teams submitted a total of 96 tractograms for scoring, we found that this crucial step of bundle segmentation was not good enough. This means that some low scores could be attributed to bad bundle segmentation during the scoring procedure, and not to problems in the input tractogram.

The data of the ISMRM 2015 Tractography Challenge^4^ is the most widely used phantom for tractography validation^1^. It is nearly the only tractography dataset with human brain geometries offering a GT. The article, published in 2017, has been cited approximately 1,000 times (as of January 2023). It has provided important insights into the challenges of tractography, particularly regarding the strong presence of false positives and the poor overlap of true positives. Now, the development of algorithms for tractography often includes a tractography validation step using this phantom.

**Figure 1:**
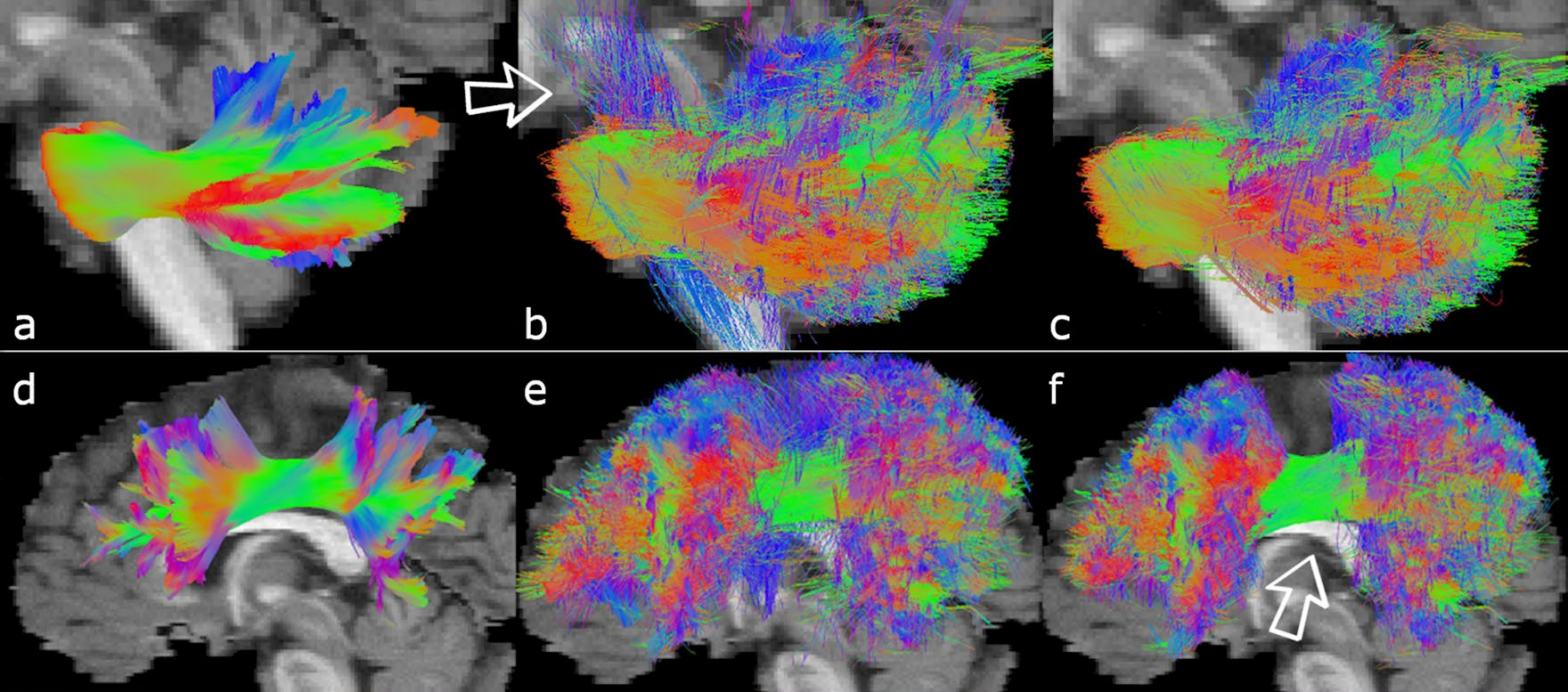
Recobundles led to poor results on some bundles. The top row shows the MCP in sagittal view. (**a**) 2015’s GT. (**b**) Streamlines recovered by Recobundles from all submissions. They include vertical streamlines that should not belong to the MCP. (**c**) Streamlines recovered using our new ROI-based segmentation. (**d**), (**e**), and (**f**) present similar patterns for the SLF.

**Figure 2:**
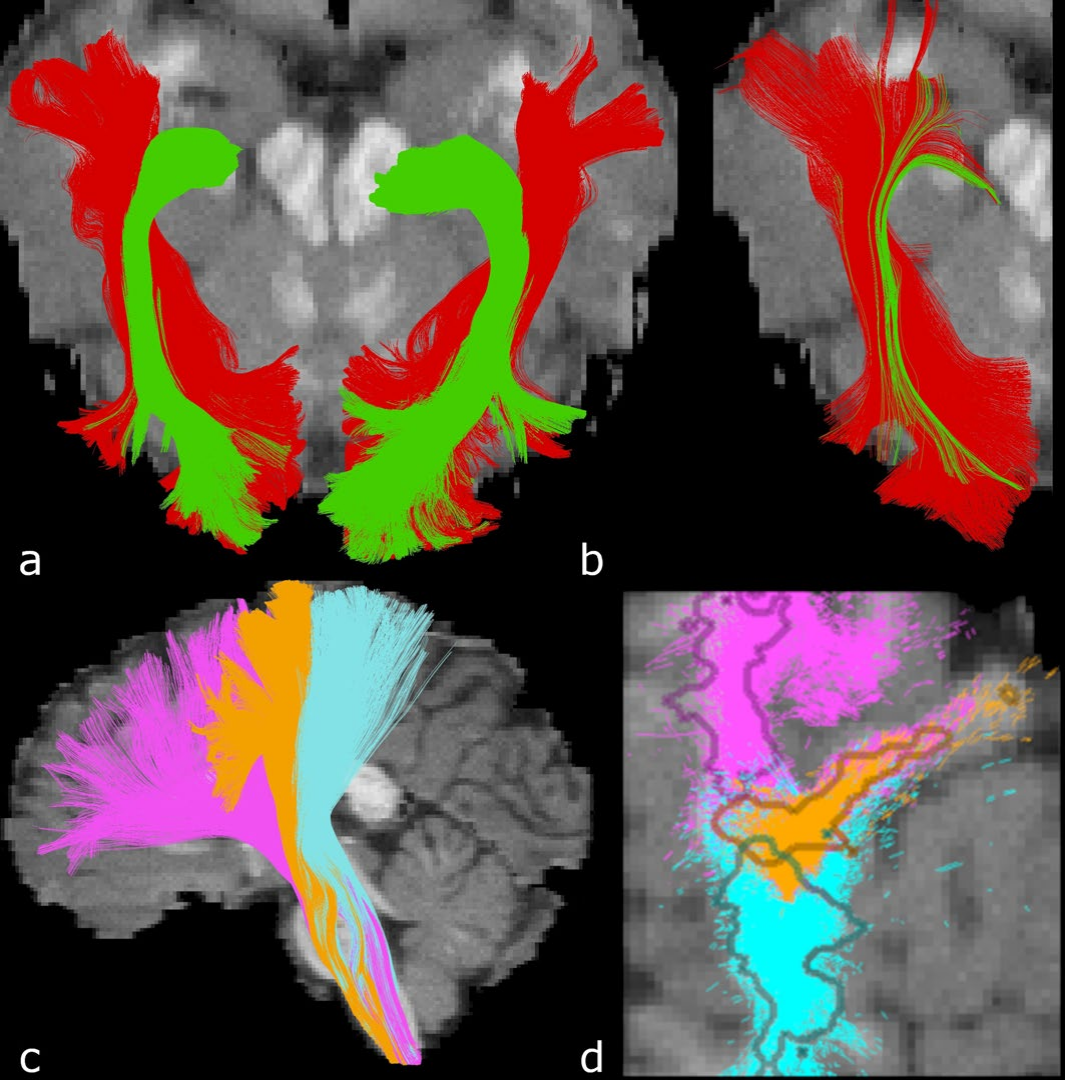
Erroneous bundle segmentation examples. (**a**) ILF (red) and OR (green) with (**b**) an example of sub-optimal bundle segmentation in submission 1.3 (using Recobundles). (**c**) FPT (pink), CST (orange), and POPT (blue), with (**d**) streamlines recovered for these bundles from all 2015 submissions. The GT bundle mask borders are shown in a darker contour. We can see that classification was sometimes arbitrary to one or the other bundle, particularly in the center.

**Figure 3:**
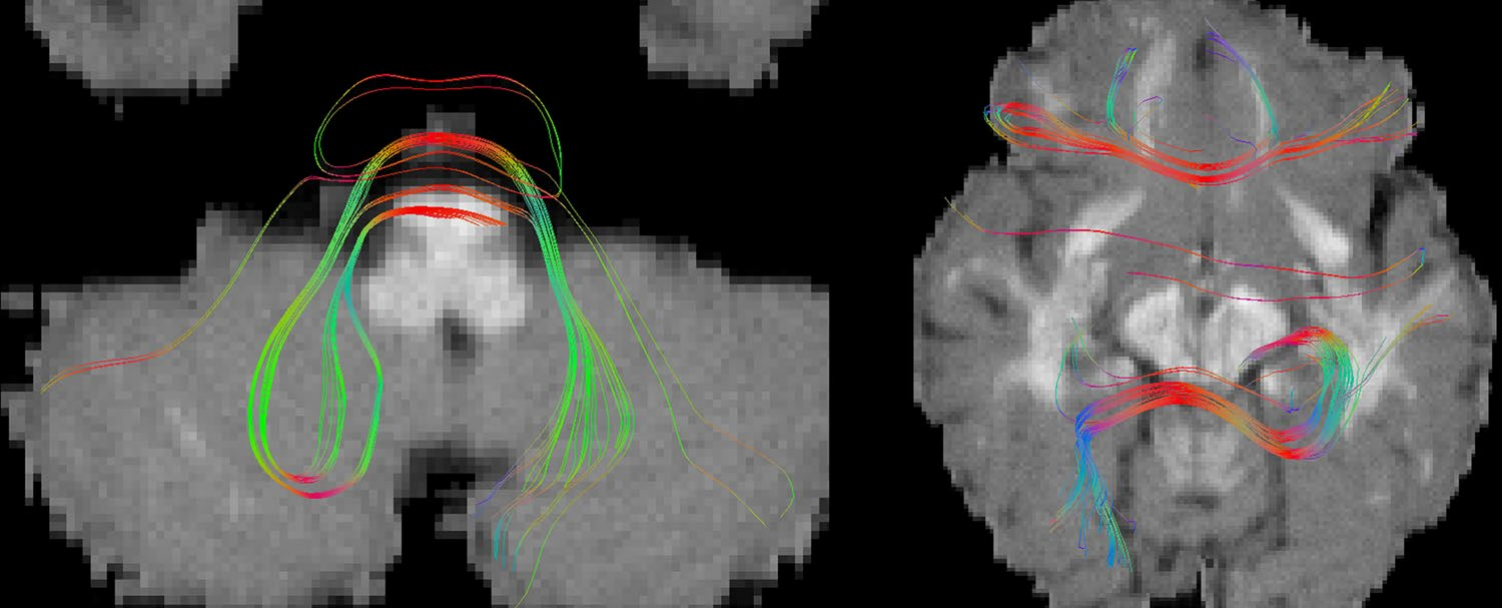
Examples of looping fibers that were hidden in the original GT tractogram.

Tractography has come a long way since its beginnings, and, generally, the most recent algorithms all achieve similar scores. Even small differences in scoring may lead to big conclusions on the choice of optimal parameters. This is particularly true in the field of machine learning in tractography^5–9^, where the validation phase often relies on final scores for fine-tuning hyper-parameters. A robust scoring system of high precision is important. In this work, we verified the quality and robustness of the challenge data and its official scoring method, including the bundle segmentation process. We identified the following shortcomings: (a) Streamlines that were clearly spurious were deemed valid (Fig. 1). (b) Some streamlines were assigned arbitrarily to the wrong GT bundle, which could lead to rapid changes in scores. Indeed, for each wrongly classified streamline, all underlying voxels are added to the overreach score. As a consequence, insights about specific bundles could be misleading. For example, similarity between FPT, CST and POPT bundles (see below for the list of acronyms) prevented a good delineation between the three bundles (Fig. 2d), leading to streamlines associated to the wrong bundle (as compared to visual assessment) and to scores that were not representative of the quality of tractography. As another example, CA and CP streamlines were sometimes not recognized because of their similarity to the CC. Finally, from Fig. 2b, one might infer that the tractography algorithm used here was unable to resolve a good spatial coverage of the OR and obtained many false positives for the ILF, but this is in fact a shortcoming of the scoring system. (c) The GT contained broken or looping streamlines, allowing such streamlines to be recognized as valid. (d) Even scoring the GT tractogram itself led to non-perfect results, with 95% overlap, 9% overreach, and a Dice score of 92%, contrary to what Fig. 3b in the original 2017 paper^4^ might suggest.

The initial segmentation procedure was based on Recobundles^10^. Each streamline in a submission was compared to the 25 ground-truth bundles and assigned to its closest ground-truth bundle based on the mean direct-flip (MDF) distance^11^, if it was within a bundle-specific threshold. Recobundles is strongly influenced by these thresholds and by the quality of the reference bundles, which contained broken and looping streamlines. Finally, it is also influenced by the ordering of bundles during the processing. In short, the previous scoring system was deemed unreliable. Here, we propose a more stable scoring system using carefully positioned regions of interest (ROIs). We present the consequences of the new process on the published scores of the 96 tractograms submitted during the challenge in 2015. Overall, general conclusions drawn in the original article^4^ still hold: most teams recovered most bundles correctly, but with lots of false positives and a poor overlap of true positives. However, individual scores for some bundles or some teams are now strongly reappraised. In particular, CA and CP are better recovered than shown in the previous analysis.

Our work also led to the production of a new ground truth tractogram with no broken or looping streamlines, revisions of the previously published scores, revisions of the initial code, and preparation of an example of well processed data. All updated data (GT, ROIs, code) and scores are available on the Tractometer website: www.tractometer.org.

## List of acronyms for bundles

BPS: Brainstem Projection System, CA: Anterior commissure, CC: Corpus callosum, Cg: Cingulum, CP: Posterior commissure, CST: Cortico-spinal tract, Fornix, FPT: Fronto-pontine tract, ICP: Inferior cerebellar peduncle, ILF: Inferior longitudinal fasciculus, MCP: Middle cerebellar peduncle, OR: Optic radiation, POPT: Parieto-occipital pontine tract, SCP: Superior cerebellar peduncle, SLF: Superior longitudinal fasciculus, UF: uncinate fasciculus.

## List of acronyms for metrics

OL: Overlap (percentage of GT voxels recovered), OR_gt_: Overreach (number of false positive voxels, normalized by the volume of the GT bundle), f1: Equivalent to the Dice score, VB: valid bundles (number of recovered bundles), VS: valid streamlines (number of streamlines in these VB), IS: invalid streamlines (number of remaining streamlines). IB: number of bundles connecting regions that should not be connected.

## Results

### Confirmation of the original scores

We first verified that we could reproduce the original results^4^ using the updated python3 version and original data. All 2015’s submissions were scored again with reviewed and updated code, with 100% reproducibility with original scores.

### Curation of the tractogram

The quality of the GT prevented the creation of ROIs. We found looping streamlines (Fig. 3) in 12 bundles (out of 25). We visually rejected broken streamlines not reaching expected regions. The biggest changes included 8% rejection in the CC, 24% and 23% rejection in both ILF and 12% and 6% for both OR. CC and right ILF included a substantial number of looping streamlines. CC had many half-streamlines stopping mid-line. ILF and OR were too similar, preventing a good segmentation; some streamlines were rejected manually. In other bundles, less than 1% of streamlines were discarded. The final clean tractogram contains 190,065 streamlines (5% rejection). We used the initial scoring system to score this curated data as a way to assess the intensity of the changes. We obtained 94% OL, 9% OR_gt_, and a Dice score of 92%.

### Creation of an ROI-based segmentation system

**Figure 4:**
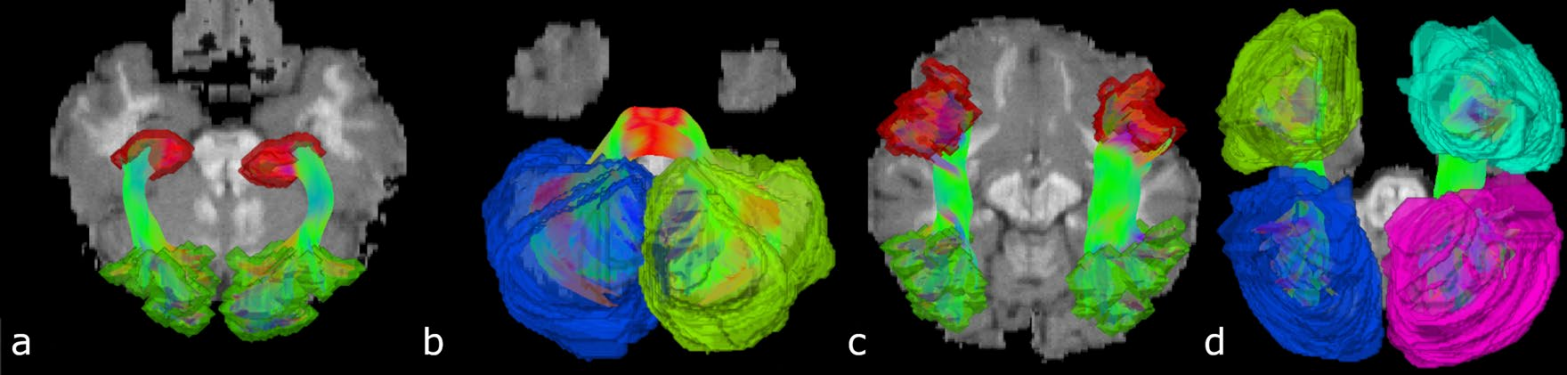
Examples of possible endpoint ROIs. (**a**) OR, (**b**) MCP, (**c**) and (**d**) SLF. Various degrees of dilation were tested. Bigger ROIs such as in B and D were necessary to score adequately all submitted tractograms.

The new segmentation relies on endpoint ROI masks and on mask_all_, and in some cases, on other criteria such as maximum length, maximum total displacement per orientation, or mask_any_:

- Endpoint masks: head and tail of the bundle. Segmented streamlines must have one endpoint in each of the two masks. Masks were created large enough to ensure they covered most variation in streamlines shape in any scored tractogram (Fig. 4).
- mask_all_: bundle envelope. Streamlines must be entirely included inside the mask. This avoids wrong-path connections, where streamlines connect the right regions but with a wrong path. Again, these masks were created as large as possible to include overreaching streamlines from most submissions.
- mask_any_: mask of mandatory passage. Streamlines must traverse it (at “any” point of the streamline).

Mask names and other criteria are included in a scoring configuration file formatted as a json file.

We verified the quality of ROIs by scoring the new curated GT data. We obtained 100% OL and 0% OR_gt_ for all bundles, as expected. When scoring the initial (non-curated) tractogram, mean OL was also 100%, with a 1% overreach, showing that modifications during curation were kept minimal. Running the new scoring system on all 96 submissions took 2h57m, vs 8h57m using the initial Recobundles-based system.

### Influence of the bundle masks on previous scores

To compare new and initial scores, we ensured that the two sets of results were indeed comparable. We noted that differences in results could be influenced by the difference in computation of the GT masks, which are called bundle masks in the original scoring data. Our new scoring was thus compared to the 2015 Recobundles system but with new bundles masks, computed with the recent definition^12^. We verified the influence of this change on the original results. Updated bundle masks led to a decrease in both OL and OR_gt_ (see Table 1), but to nearly unchanged f1 scores (*p*-value > 0.1). To compare with the new scoring system, which scores 21 bundles, these results were computed over 21 bundles using the mean value of FPT/POPT/CST.

**Table 1:** Impact of updated bundle masks on the scoring, using the original Tractometer scoring system.

Mean	Original 2015 scores (old bundle masks)	Updated 2015 scores (new bundle masks)
OL (%)	35.6 ± 16.5 [1.1 to 76.6]	34.7 ± 16.2 [1.1 to 75.4]
OR_gt_ (%)	29.0 ± 25.9 [1.0 to 152.5]	25.5 ± 23.3 [0.9 to 137.7]
Dice / f1 (%)	37.8 ± 12.6 [2.0 to 56.1]	37.8 ± 12.8 [2.0 to 58.0]

### Influence of the new scoring system on scores

Visually, new scoring of the initial 2015 submissions led to better segmentation (see Fig. 1). On average, Dice scores were significantly different (*p* < 0.001) (see Table 2), but with an average change of only 2%, offering similar rankings. The biggest variations included an upgrade of 9 places for submission 17.0 and a drop of 13 places for submission 1.4. Top 8 submissions stayed the same but in a different order, as did the bottom 8 submissions. The average absolute difference was 2 positions out of 96, thus leading to similar conclusions as in the original analysis. However, some bundles showed major differences (see Table 3) in scores and in ranking**.** The detailed score tables for each team, each bundle is provided on the website.

**Figure 5:**
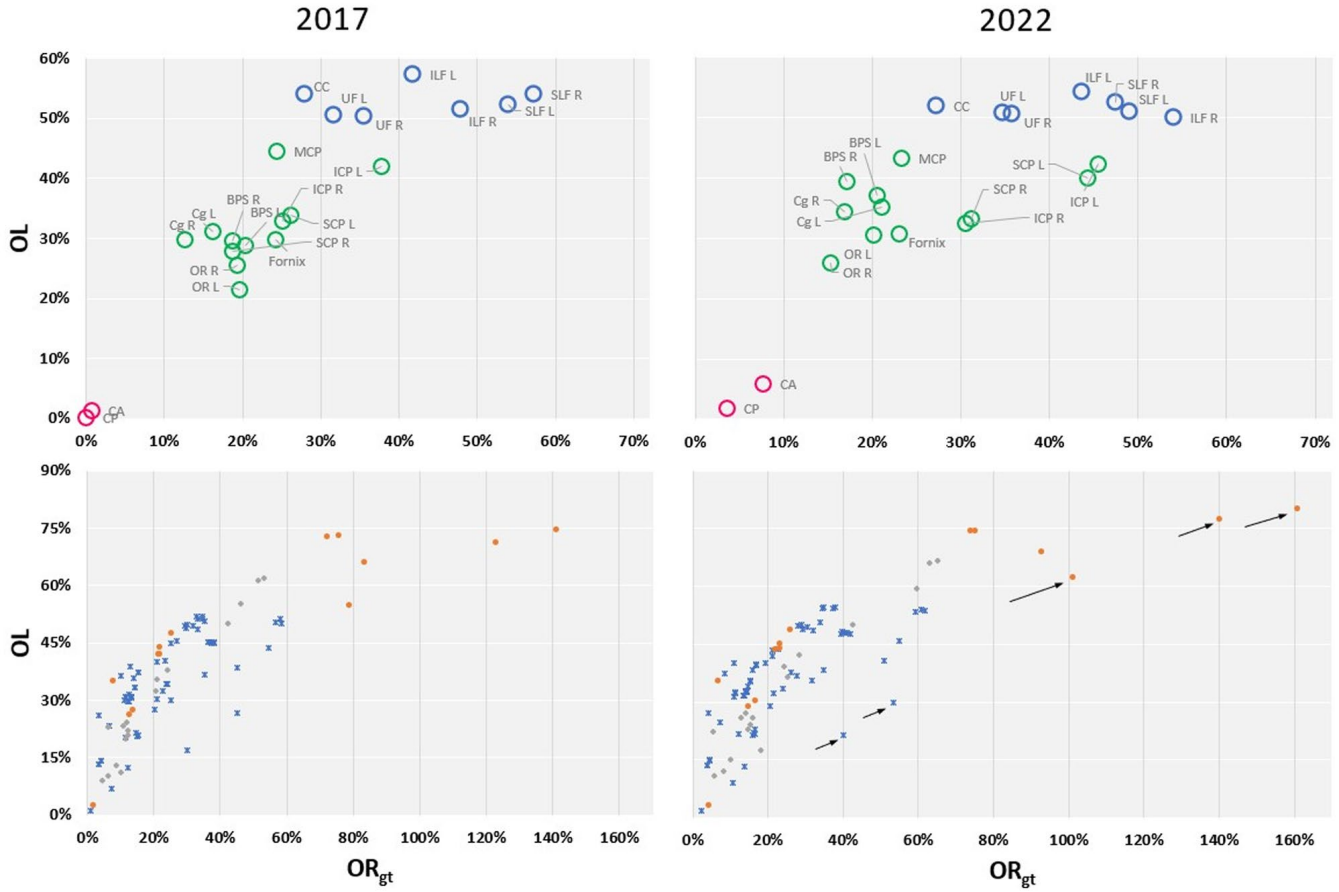
Overlap (OL) vs Overreach (OR_gt_) scores in 2022 versus 2015 (with updated masks). Best results should have high overlap (top) and low overreach (left). Top graphs: scores per bundle (averaged over all teams). Colors reflect the differences between easy (blue), average (green) and hard-to-track (pink) bundles, as in^4^. Bottom graphs: scores per submission (averaged over all bundles). Colors reflect the algorithm choice: deterministic (blue), probabilistic (orange) or others (gray). Arrows highlight the displacement of some submissions with strongly changed scores (generally, better OL, worst OR_gt_).

- VB: As seen in Table 3, CP and CA were discovered more often than estimated in the original analysis. They still are the two most difficult bundles to recover, but to a lesser extent.
- VS: Biggest change in VS is seen in the CC, partly because it is by far the biggest bundle. When observing the VS in raw numbers rather than as percentages of the total number of streamlines, a comparison between the two scoring systems reveals drastic changes, as seen in Table 3.
- Bundle coverage: Fig. 5 (top section) compares the bundle dispersion in OL and OR_gt_ between the two scoring systems. Main changes are reported in Table 3. Overall, f1 score was improved, particularly for the two BPS bundles and for the left OR, for which modifications have been brought in the GT data. Bottom section in Fig. 5 compares the submissions dispersion for these metrics. Overall, previous conclusions still hold: probabilistic tracking may help generate highest OL, but with highest OR_gt_. Submissions 9.1 and 9.2 (best OL) only obtain Dice scores of 45% and 46%, placing them in 43^rd^ and 38^th^ rank.

**Table 2:** Effect of the new segmentation on average scores. Nb: Number of submissions who recovered the bundle.

Mean	Updated 2015 scores (21 bundles)	New scores
VB	18.0 ± 2.7 [5 to 20]	18.5 ± 2.3 [9 to 21]
Nb	82.1 ± 25.4 [2 to 96]	84.5 ± 20.8 [22 to 96]
VS (%)	53.6 ± 23,5 [3.7 to 92.5]	52.5 ± 22.1 [4.3 to 88.6]
OL (%)	35,7 ± 16.0 [1.3 to 74.3]	37.8 ± 16.4 [1.8 to 80.0]
OR_gt_ (%)	26.7 ± 23.7 [1.1 to 141.4]	29.1 ± 26.7 [2.4 to 161.1]
Dice / f1 (%)	38.4 ± 12.1 [2.4 to 54.9]	40.7 ± 12.2 [3.1 to 57.9]

**Table 3:** Effect of the new scoring: some of the main changes in specific bundles (average over teams).

Mean	Bundle (L/R = left / right)	Tractometer 2015 (21 bundles)	Tractometer 2022	Difference
Nb submissions recovering the bundle	CP: CA: SCP L/R:	2 12 86 / 83	25 22 88 / 88	+ 23 + 10 + 2 / + 5 Others: Differences in less than 4 submissions
VS (Total number of streamlines recovered amongst all teams)	CP: CA: SCP L/R: Cg L/R: BPS L/R: OR L:	2 64 38,193 / 23,607 278,422 / 238,027 322,645 / 520,016 49,883	172 2011 59,109 / 36,171 374,647 / 375,725 437,459 / 636,523 65,161	+ 8,500% + 3,400% + 55% / + 53% + 35% / + 58% + 36% / + 22% + 31% Others: Less than 20% variation
OL (%)	BPS L/R: OR L: SCP L/R:	28.8 / 29.7 21.4 33.9 / 27.9	37.1 / 39.4 30.6 40.0 / 33.2	+ 8.3% / + 9.7% + 9.1% + 6 / + 5 Others: Less than 5% variation
OR_gt_ (%)	SCP L/R: SLF L/R: ICP L/R: CA: ILF R:	26.1 / 18.8 50.4 / 57.3 37.8 / 25.1 0.7 41.8	44.3 / 31.2 49.0 / 47.5 45.5 / 30.5 7.7 54.0	+ 18.2 / + 12.4 -5.0 / -9.8 + 7.7 / + 5.4 + 6.9 + 6.2 Others: Less than 5% variation
Dice / f1 (%)	BPS L/R: OR L: CA:	34 / 36 25 2	44 / 47 37 7	+ 10 / + 11 + 12 + 5 Others: Less than a 3% variation

### Usage on new data

We successfully used the Tractoflow pipeline^13^ with the noisy data using both the particle filtering tractography (PFT tracking) and the local tracking algorithms to obtain two full tractograms that were scored with the new system. The PFT version led to the best Dice score (64%. Previous best was 58%), with an average OL and OR_gt_ of 76% and 60%. The local tracking version, which used a dilated white matter (WM) mask, obtained the best overlap (91%. Previous best was 80%), but with more OR_gt_, explaining its lower, yet high, Dice score (57%).

## Discussion

We have developed an enhanced Tractometer scoring system for the ISMRM 2015 Tractography Challenge data. It uses carefully determined regions of interest. It offers more reliable results because the segmentation now depends only on the quality of the ROIs. It does not depend on other aspects that were important in the Recobundles segmentation, such as the ordering of the bundles, quality of the reference tractogram (recovered bundles could hide broken/looping streamlines because the GT data itself contained such streamlines), and threshold values for the MDF. In short, our new segmentation is strict enough to prevent the inclusion of noisy streamlines but flexible enough to allow scoring submissions of varied streamline lengths, curvature, fanning, and tracking masks.

Overall, the new segmentation offers similar rankings as before when using averaged values over all bundles and all teams, but scores for some bundles were strongly modified.

### Verification of the original code

No error was found in the original code. Importantly, however, tractogram formats and headers management has evolved significantly since 2015. Users should verify that their tractogram are correctly interpreted when using the updated python3 code.

### Verification of the original scores

The scores published in the 2017 article were good, but the detailed scores published on the website contained errors which are now corrected. Please also note that some wrong numbers tend to be relayed amongst publications citing the ISMRM challenge results. We urge readers to rely on the up-to-date scores currently published on the official website (www.tractometer.org).

We also brought modifications to metrics terminology to avoid confusion:

1. VS/IS: In the original analysis, the term “connection” was used in the terms valid/invalid/no connections (VC, IC, NC). However, VC was defined as the number of streamlines belonging to a valid bundle and could actually include broken or prematurely stopped streamlines that do not reach any gray matter region as long as they were classified as belonging to the bundle by the chosen segmentation process. The word may encourage wrong interpretation of the results, suggesting that they measure connectivity between brain regions. We renamed VC as VS (valid streamlines). We regrouped IC and NC under the term IS (invalid streamlines).
2. IB: Segmenting invalid streamlines into invalid bundles gives insight on typical errors recovered recurrently over multiple submissions, but their number (IB) may however be misleading as a scoring metric because it depends on the definition of these bundles. The number of invalid bundles obtained with Quickbundles depends strongly on the type of invalid streamlines. Segmentation of spurious streamlines with varied shapes and distribution offers scores that are difficult to interpret. Even a few misplaced streamlines may lead to a rapid increase in IB, which should not be used to infer the quality of the scored tractogram. The IB score should be used with great care. IB scores are therefore not used anymore in our work.

### Curation of the data

We removed streamlines that prevented the creation of a good scoring system. Curation of the data was kept as minimal as possible, leading to the same Dice score as with the original GT (with the original scoring). Still, the new GT now corresponds less perfectly with the associated DWI. Creating a new simulated DWI with Fiberfox^14^ would be possible, but future work using this new data could not be compared with the scores presented here from teams who participated in the challenge.

One note to the reader should be made here. The phantom was created with knowledge available at the time. Although the bundles have names that correspond to known anatomical tracts, users should keep in mind that they might not present exact characteristics and features compared to the real tracts^15^. These bundles should be used as phantom parts, not as anatomical references. Here is a short list of differences that were noticed between the GT bundles and known anatomical landmarks.

- CC: The corpus callosum is known to contain a majority of homotopic connections^16^. Heterotopic connections do exist, but are less documented^17^. Many heterotopic connections are found in this GT (ex, ventro-striatal).
- Cg: The Cg consists of 5 sub-bundles^18^. The GT bundle lacks the posterior part (named CB-V in the paper).
- ICP: This bundle should end in the brainstem, but the GT bundle contains two sub-bundles; one is anatomically correct but the other, looping back into the cerebellar cortex, does not correspond to any known path in the human anatomy.
- OR: The current bundle would be better named as thalamo-occipital connections. The OR is typically defined as the streamlines from the peri-calcarine fissure to the thalamus^19^, but in this GT, the bundle extends to a larger section of the occipital lobe. Note also that the Meyer's loop^20^ is absent from the current GT.
- ILF: The ILF should reach the anterior temporal lobe^21^. However, in the initial version of the phantom, it reached a larger region, extending posteriorly close to the (expected) Meyer’s loop region. This was modified in the new curated data and therefore the ILF is now more anatomically reliable.
- UF: As of 2018^22^, the uncinate fasciculus is now considered with a larger fanning both anteriorly in the frontal cortex and posteriorly in the temporal cortex.
- CST / FPT / POPT: These three bundles appear intricate, but should be more different. The cortical terminations of the CST should be constrained to the precentral and postcentral gyri^23^. Both FPT and POPT should end in the pons, but the bundles go further down, nearly to the medulla (see Fig. 2).

Due to these differences, the ROIs defined here do not represent perfect anatomical features either, but are only the necessary tool to segment bundles before the scoring.

Creating new bundles with better anatomical features would require developing a new simulated DWI data, i.e., a new phantom. This, as stated above, was not the objective of this work. We encourage the community to produce new and varied phantoms as there is a lack of validation data in the field of tractography. However, here, the goal was essentially to improve the existing one and allow, particularly, the machine learning community to adequately compare their results with previous state-of-the-art tractography tools. We present in a section below conclusions and suggestions drawn from our analysis to readers interested in proposing a new phantom.

### Preparation of the new segmentation technique

To allow for a good bundle segmentation in the submitted data of most teams, the endpoint ROIs had to be created very large, sometimes up to a 16-pass dilation of the GT bundles’ endpoint ROIs, and up to an 11-pass of the bundles’ mask_all_. This could reveal that the stopping criteria was not well defined in many processing pipelines. It generally depends on a WM mask, which may come either from a thresholded FA map (typically ~ 0.1 to 0.2) or from segmentation from the T1. In the first case, the simulated DWI may have acted differently than usual and provided FA values that would require a different threshold. In the second case, the T1 is also simulated. Segmentation algorithms were not created to deal with “fake” images and may have resulted in WM masks of lesser quality. We consider that the goal of this challenge was to evaluate the ability of tractography algorithms to understand diffusion information and to follow diffusion anisotropy information through challenging paths such as fiber crossing and bottlenecks. We have decided not to penalize submissions with streamlines going further than expected. For instance, some submissions had streamlines from the OR going out of the thalamus without stopping or streamlines from the Fornix looping very far off the mamillary bodies, or even streamlines going out of the brain. Our ROIs thus spill out of realistic anatomical regions in an attempt to include the biggest part of every submission’s bundles. We can still segment bundles correctly by combining the endpoint ROIs with the mask_all_. Of note, this prevented an adequate segmentation of IB. Considering that we did not use this score for the original version either, IB was simply not included in our analysis.

### Analysis of the score differences

Compared to the initial analysis^4^, it is still true that teams were able to recover most bundles. It is also still true that, on average, only half of the streamlines in the submitted tractograms are valid streamlines. Finally, we still find that probabilistic tracking may help generate the highest OL, but with the highest OR_gt_ when compared to deterministic tracking, resulting in small changes on the Dice score.

*VB*: CA and CP are still the two most difficult bundles to reconstruct, but although they are still a well-defined category in Fig. 5, it is to a lesser extent. Using Recobundles, CP was scored after CC; these streamlines were often associated to the CC and thus ignored when segmenting the CP. Other changes in recovered bundles are explained by the fact that newly found bundles generally contained only a few very small streamlines that may be harder to compare with reference streamlines using the MDF metric (in Recobundles). The hard-to-track and medium-difficulty bundles (Fig. 5) are now less separated categories.

*VS/IS:* Often, the additional recovered streamlines were of very poor quality, and other metrics were not improved much. The total percentage of VS, averaged over all teams, all bundles, only varied by less than 1%. Yet, it represents an average of 1000 streamlines per submission. In the future, with algorithms becoming ever better and researchers trying to push the limits of tractography, these small differences in scoring could impact researcher choices in implementation.

*Bundle coverage*: Despite the big changes in the total number of recovered streamlines in individual bundles throughout the 96 submitted tractograms, general scoring metrics stayed similar, but ranking amongst teams was modified.

### Suggestions for the creation of a new phantom

The final comparison of “winners” based on the Dice score, either in the original analysis or here, did not allow for a clear definition of the best tractography parameters. This can be explained by the large influence of preprocessing steps such as the choice of tracking space, the tracking masks, the registration quality, and so on. Future phantoms should limit the possibilities to ensure that they can understand specifically our ability to follow diffusion information in the brain, in other words, the “tracking” aspect, rather than the quality of the whole pipeline. We present some afterthoughts here.

1. The level of complexity in the challenge data was good. It presented human-like geometries with multiple bundle crossings or bottlenecks. Its number of bundles was good and allowed for the creation of a scoring system.
2. The associated simulated T1 data, however, was not realistic enough to obtain good results in segmentation software such as Freesurfer^24^ or FSL FAST^25^ for instance. We suggest that future work should include a list of potentially interesting masks, particularly a WM mask that could be used as a tracking mask.
3. The quality of individual streamlines, not only of bundles as whole entities, should be verified, both in the GT and during scoring.
4. Developers should specify a way that users may verify their tractogram format to prevent shifts (ex: ± 0.5 when the origin of a voxel coordinate is considered at the center or at the corner of the voxel) or swapping of axes during interpretation (ex, specifying the orientation).
5. Developers should specify in which space the final scoring will be performed. Users applying a substandard registration between T1 and DWI spaces could be strongly disadvantaged, even if their tracking algorithm itself was perfect.
6. OL, OR_gt_, Dice scores offer good insights, but there is still a lack of metrics comparing the shape of individual streamlines in the literature that should be addressed.

### Quality of the Tractoflow-processed data

The data was processed using state-of-the-art tools and presented a Dice score of 65%, which none of the 96 submissions was able to achieve in the ISMRM 2015 challenge. Improvements seem to come from a combination between better preprocessing (distortion correction, registration), improved masks, and tracking algorithm/parameters. However, at this time, it is impossible to compare our preprocessing pipelines to the 2015 teams’ pipelines defined in the supplemental file of the original paper^4^, considering that we only have access to resulting tractograms, and not their preprocessed diffusion data. When supervising our pipeline, we noticed that using the additional-reversed phase b0 to perform top-up correction led to a better preprocessed DWI, visually (without it, Dice score dropped to 57% for the PFT-tracking). We also carefully supervised the WM mask’s dilation to ensure its similarity to the GT bundles. This mask is used as seeding mask for both tracking methods, and as tracking mask for the local-tracking. Finally, the PFT-tracking version of Tractoflow uses a tracking algorithm that existed in 2015 but was not used by any team. A more in-depth understanding of the impact of each preprocessing step and parameter value would be out of the scope of this paper. Future work performing a systematic Tractometer-like^3^ approach should be done to analyse the impact of every processing step of tractography pipelines.

### The phantom as a training set?

This scoring system is a validation technique much used in machine learning (ML) studies, as mentioned above. One difficulty is that the DWI is not perfectly similar to a real, non-simulated diffusion data. Good practice in ML would recommend a clear definition of a training set, validation set, testing set. Due to the unique properties of the phantom, it may prove difficult to train algorithms on real data and hope for good results at validation time on the simulated data. In practice, teams seem to avoid training on the ground truth bundles by training on tractograms produced in-house on the phantom. Then, differences in scores between ML algorithms may arise from the differences in the quality of the training data. Therefore, we also offer the Tractoflow-processed data in open access on the website, including the preprocessed DWI and tracking masks. It could be used as common training data for all teams.

## Conclusion

We proposed a new and enhanced Tractometer scoring system based on manually defined regions of interest rather than on bundle recognition. Bundles are now more reliably segmented, offering more reliable metrics for future users of this phantom and its scoring system. We provide on the Tractometer website all necessary tools for a robust scoring of any new tractogram with our new scoring system: the ROIs and configurations files necessary to run the code, the tables of detailed results and the Tractoflow-processed data.

This should help researchers better develop and evaluate the next generation of tractography algorithms.

## Methods

### Verification of the original scores

The original code was converted to python3, proof-read and reviewed to ensure it was still suitable with today’s standard. Metrics terminology was revised. All 2015’s submissions were scored again.

The original code included forced shifting (adding 0.5 values) of .trk (trackvis) files. In the updated code, tractograms are simply loaded through dipy’s load_tractogram method^26^. No further verification is performed on the validity of space attributes.

### Curation of the GT tractogram

The GT bundles were modified to enable the creation of the ROIs. Analysis of the GT tractogram revealed short/long, looping, and broken streamlines. Streamlines from the GT bundles were filtered to keep only those with length in the range 20–200 mm (generally streamlines presenting looping shapes) or recovered as loops using scilpy were discarded (see https://scilpy.readthedocs.io/). Other streamlines were discarded based on visual analysis of the bundles. Rejection was kept as small as possible to ensure good compatibility between the tractogram and the associated simulated DWI. CST, POPT, and FPT were too similar and difficult to segment adequately (Fig. 2) and were gathered into a new bundle called Brainstem Projection System (BPS). The ILF and OR were also too similar, preventing a good segmentation (Fig. 2), either with Recobundles or with ROIs. In this case, we chose to filter out some streamlines to better separate the two bundles. To assess the intensity of these changes, we scored this curated GT with the original scoring system.

### Creation of a ROI-based segmentation system

All of the masks were created by looking carefully at both the GT data and the general distribution of results from the tractograms submitted to the Challenge in 2015.

Endpoint ROIS: GT streamlines’ endpoints were saved as head and tail masks. We then dilated these two masks (11-pass on average, see Fig. 4). Some endpoint ROIs were modified manually based on visual inspection of results. Examples of modification were: dilation to reach the end of the cortex in some regions, manual dilation of the OR’s ROI to include more of the thalamus without spilling into the ILF, manual separation between hemispheres, careful separation of anterior/posterior ROIs in the case of the cingulum and of the fornix. The CC was separated into sub-bundles for segmentation purposes (CC_u_shaped, CC_ventro_striatal1, CC_ventro_striatal2, CC_temporal), allowing for a better delimitation of endpoint ROIs. However, only the total CC, composed of the re-merged sub-bundles, is used during scoring. Similarly, the ICP was segmented into ICP_part1 (similar to its anatomical definition) and ICP_part2 (looping back into the cerebellar cortex).

mask_all_: GT streamlines paths were saved as binary masks and dilated (by default, the number of passes was 3 but some bundles required varied parameters, up to an 11-pass for the CC). These GT masks were combined with both endpoint ROIs for each bundle. Manual modifications were also applied, generally more manual dilation.

mask_any_: They were defined using manually positioned boxes of interest.

### Influence of the bundle masks on scores

To compare new and old scores, original bundle masks were computed again using more recent technology. As suggested in 2017 by Rheault et al.^12^, bundle masks should not recover only voxels containing streamlines points (even after resampling), but should rather account for the whole segment between two points. We computed the new masks with scilpy. Bundles segmented using the Recobundles-based system were scored again using the same metrics but with the new GT bundle masks. Final Dice scores, averaged over all bundles, were compared to previous scores using a Student T-test.

### Influence of the new scoring system on scores

Newly segmented bundles of the 96 submissions were scored using the same metrics as before. Again, final Dice scores, averaged over all bundles, were compared to previous scores using a Student T-test.

### Usage on new data

We prepared a new tractogram to be scored using recent state-of-the art techniques. The tractogram was prepared by running the Tractoflow pipeline^13^ on the noisy DWI, using the version with additional reversed b0 to allow for topup correction. The pipeline was modified to skip the N4 denoising step on the T1 data, which produced irregular results, probably due to the fact that a T1 is in fact a simulated dataset. Two tracking algorithms were tested. First, PFT tracking on WM maps. Second, local tracking on a mask of WM that was first modified to pass visual quality check: it was eroded (1-pass) and dilated again (2-pass). Both versions were scored using the new system.

## Data Availability

The datasets generated and/or analysed during the current study are available on the Tractometer website: www.tractometer.org.
